# Œsophagite disséquante: une cause rare de dysphagie

**DOI:** 10.11604/pamj.2014.17.164.4039

**Published:** 2014-03-06

**Authors:** Houda Meyiz, Ihsane Mellouki

**Affiliations:** 1 Université de Sidi Mohammend Ben Abdellah, Faculté de Médecine et de Pharmacie, Département de Gastroentérologie C, Fès, Maroc

**Keywords:** Œsophagite disséquante, dysphagie, AINS, esophagitis dissecans, dysphagia, NSAIDs

## Image en medicine

L'œsophagite disséquante chronique (ODC) est une affection rare, de découverte récente. Elle est la conséquence d'altérations dégénératives de la couche superficielle de la muqueuse œsophagienne, réduisant l'adhésion entre les cellules de l'épithélium malpignien. Les ODC sont primitives dans 20% des cas. Les ODC secondaires surviennent dans un contexte de RGO, de maladie dermatologique (lichen plan, pemphigus vulgaire), d'œsophagite à éosinophiles, de prise médicamenteuse (biphosphonates, AINS) ou de maladie cœliaque. Cette affection se manifeste cliniquement par une dysphagie chronique, plus rarement des douleurs thoraciques et un amaigrissement. A l'examen endoscopique, la muqueuse est blanchâtre, marquée par des stries annulaires transversales et qui se détache en lambeaux spontanément ou lors des biopsies. Il existe des sténoses souvent annulaires. La principale lésion anatomopathologique est un clivage épithélial, habituellement sous la membrane basale, mais sans infiltrat inflammatoire, ni hyperkératose. L'évolution est chronique. Il n'existe aucun traitement spécifique. Nous rapportons le cas d'une patiente âgée de 44 ans, sans antécédents pathologiques notables, qui présente depuis 1ans une dysphagie aux solides. L'examen clinique et les examens biologiques de routine étaient sans particularité. L'endoscopie oesogastro-duodénale a objectivé la présence d'une sténose serrée du tiers supérieur de l'œsophage, dilatée sans complications aux bougies de Savary, avec individualisation en aval de la sténose d'anneaux translucides avec une muqueuse qui se dilacère aux biopsies. L'étude anatomopathologique était en faveur d'une œsophagite non spécifique, et le clivage intraépithélial n'a pas été mis en évidence. L'évolution a été marquée par la récidive de la symptomatologie ayant nécessité deux séances de dilatation supplémentaires.

**Figure 1:**
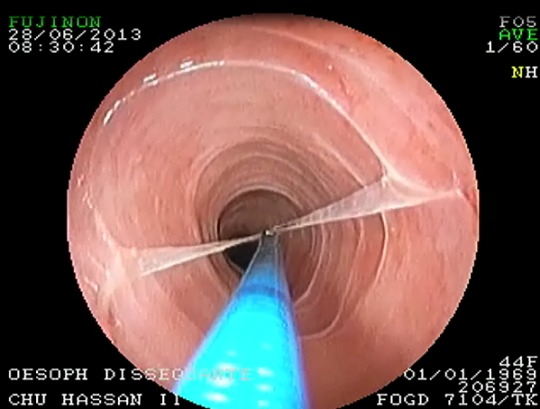
Aspect endoscopique objectivant des anneaux translucides avec une muqueuse qui se dilacère aux biopsies

